# Centripetal Acceleration Reaction: An Effective and Robust Mechanism for Flapping Flight in Insects

**DOI:** 10.1371/journal.pone.0132093

**Published:** 2015-08-07

**Authors:** Chao Zhang, Tyson L. Hedrick, Rajat Mittal

**Affiliations:** 1 Department of Mechanical Engineering, the Johns Hopkins University, Baltimore, Maryland, United States of America; 2 Department of Biology, University of North Carolina at Chapel Hill, Chapel Hill, North Carolina, United States of America; Brown University, UNITED STATES

## Abstract

Despite intense study by physicists and biologists, we do not fully understand the unsteady aerodynamics that relate insect wing morphology and kinematics to lift generation. Here, we formulate a force partitioning method (FPM) and implement it within a computational fluid dynamic model to provide an unambiguous and physically insightful division of aerodynamic force into components associated with wing kinematics, vorticity, and viscosity. Application of the FPM to hawkmoth and fruit fly flight shows that the leading-edge vortex is the dominant mechanism for lift generation for both these insects and contributes between 72–85% of the net lift. However, there is another, previously unidentified mechanism, the centripetal acceleration reaction, which generates up to 17% of the net lift. The centripetal acceleration reaction is similar to the classical inviscid added-mass in that it depends only on the kinematics (i.e. accelerations) of the body, but is different in that it requires the satisfaction of the no-slip condition, and a combination of tangential motion and rotation of the wing surface. Furthermore, the classical added-mass force is identically zero for cyclic motion but this is not true of the centripetal acceleration reaction. Furthermore, unlike the lift due to vorticity, centripetal acceleration reaction lift is insensitive to Reynolds number and to environmental flow perturbations, making it an important contributor to insect flight stability and miniaturization. This force mechanism also has broad implications for flow-induced deformation and vibration, underwater locomotion and flows involving bubbles and droplets.

## Introduction

Insects and other flying animals support themselves in the air using flapping wings which rely on aerodynamic mechanisms different from those found in man-made rotary and fixed wing configurations. Moreover, the unsteady aerodynamics of beating insect wings typically provide greater coefficients of lift than these conventional wings, facilitating phenomenal flight performance over scales ranging from a few millimeters to tens of centimeters [1]. Investigation of these unsteady mechanisms and associated flow features has a long history of ingenious experiments [2–5], which have identified the leading-edge vortex (LEV), as well as clap-and-fling [6], wing rotation [7] and wake-capture [8]. However, the net aerodynamic force on the wing is given by

$$\begin{array}{c} {\vec{F}}_{B}=\int_{B}(p\hat{n}+{\vec{\tau }}^{w})dS\;\text{for}\;\;i=1,2,3 \end{array}$$ (1)

where *B* is the surface of the wing, $\hat{n}$ the unit normal pointing into the body, and *p* and ${\vec{\tau }}^{w}$ are the surface pressure and surface shear (all symbols used in the paper can be found in S1 Text), respectively, and experiments are limited in their ability to measure the surface pressure and shear. This, coupled with the difficulty of simultaneously measuring the velocity and vorticity in the three-dimensional flow-field has limited the extent to which individual contributions of various mechanisms can be precisely delineated via experiments. Consequently, a clear understanding of the aerodynamic mechanisms that generate the lift required to stay aloft, remains elusive.

Computational fluid dynamic (CFD) modeling provides the quantities needed for a clear delineation of force production but a conventional analysis of pressure and viscous shear on the wing surface via Eq (1) does not distinguish the partial contributions of the different unsteady mechanisms and flow features to the pressure on the surface. Doing so is non-trivial since pressure in an incompressible flow is governed by an elliptic equation and is therefore coupled to the flow velocity at every point in the flow field.

Here we construct a formulation of the total force exerted by an incompressible Newtonian flow on an immersed body that clearly delineates the partial contributions of various physical mechanisms and features to the total aerodynamic force. This force partitioning method (FPM), the derivation of which is described in S2 Text, is implemented into a three-dimensional, sharp-interface immersed boundary Navier-Stokes solver [14]. Using it, we are able to partition the total instantaneous aerodynamic force on a flapping wing into components associated with wing kinematics, vorticity, and viscous shear and dissipation.

**Fig 1:**
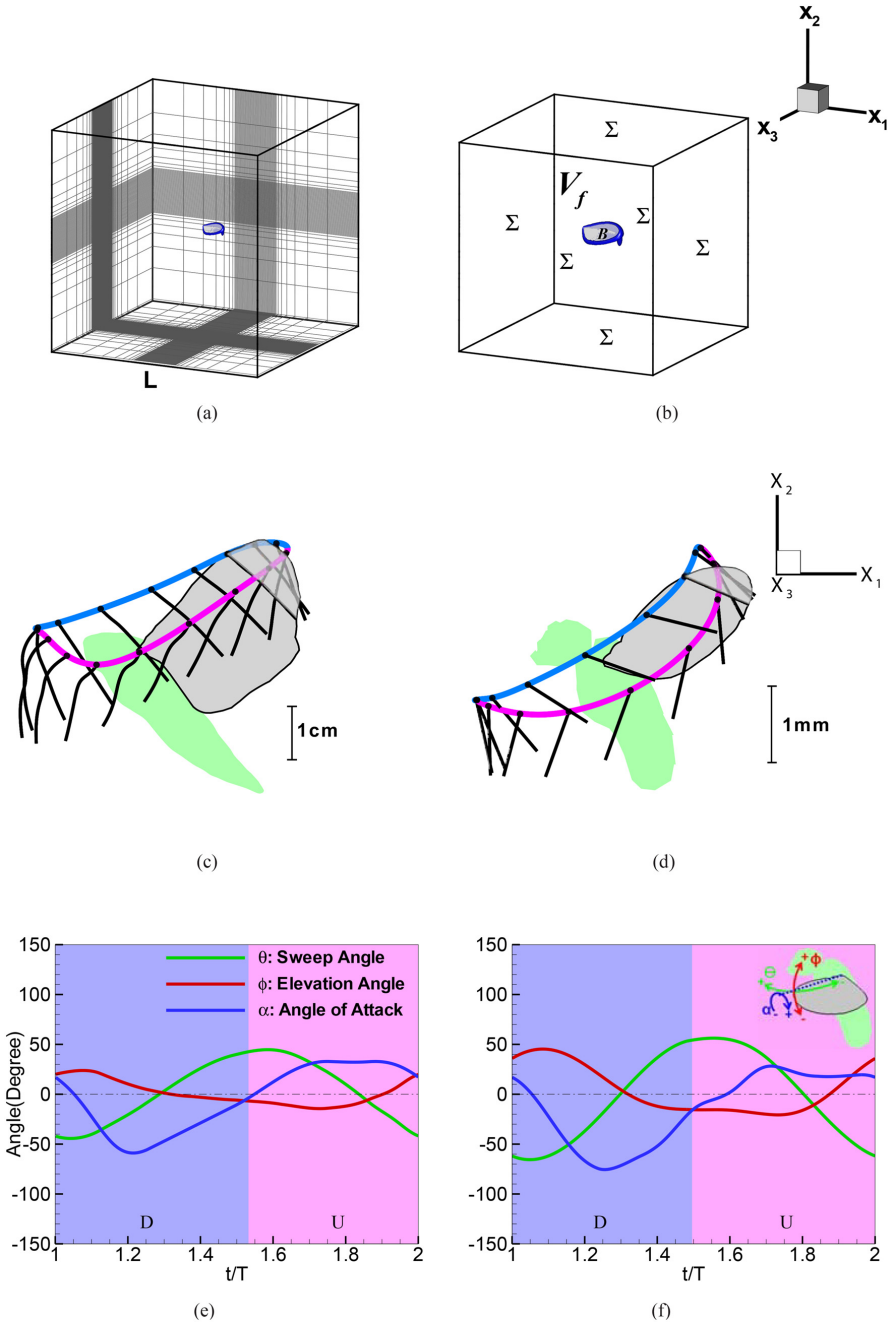
(a) Typical non-uniform Cartesian mesh employed in the simulations. (b) Schematic of the control volume (not to scale) employed for the simulations and FPM. (c) Kinematics of the wing of the hovering hawkmoth and (d) fruit fly. In these plots, the trajectory of the leading edge of the wings at 2/3 span is identified by a thick line which is blue during downstroke and pink during upstroke. The chordlines at 2/3 span are also identified by black lines with circular “heads”. Time series of three characteristic angles (see inset in (f)) that define the wing kinematics for the (e) hawkmoth and (f) the fruit fly. For the hawkmoth, the instantaneous 3D wing shape and kinematics were quantified via high-speed stereo videogrammetry from recordings of the animal hovering steadily in front of an artificial flower [9]. For the fruit fly, a flat-plate wing was constructed from a high-resolution image of a fruit fly wing. Flapping kinematics consisting of three angular degrees of freedom were then extracted via high-speed stereo videogrammetry of a fruit fly, hovering shortly after takeoff, and imposed on the wing, resulting in rigid wing flapping kinematics. Fruit fly wings exhibit little deformation and the use of rigid wing kinematics is typical of mechanical [10] and recent computational [11–13] models of their flight.

The formulation is applied to the flapping wings of a hovering hawkmoth (*Manduca sexta*) and a fruit fly (*Drosophila melanogaster*) in slow climbing flight to reveal similarities and differences in the mechanism of lift production in these insects. Besides the dissimilarities in wing kinematics and the shape and deformation characteristics of the wings (Fig 1), the two flyers operate in Reynolds number regimes separated by over an order of magnitude. A comparative analysis of these two cases therefore provides an excellent substrate for investigating the scaling of lift force.

Our analysis conclusively shows that while the LEV is the dominant mechanism for lift generation in these flapping wings, there is an additional mechanism, the “centripetal” acceleration reaction, that provides a significant contribution to the total lift force. This force component is completely determined by the centripetal acceleration of the wing which requires rotation with associated tangential motion, and to to our knowledge, has not been identified in any past study of flapping flight. This component of lift is independent of Reynolds number and environmental disturbances; it therefore represents an effective and robust mechanism for weight support at all scales. The implications of this mechanism go far beyond insect flight since it could also play an important role in a wide variety of vortex dominated flows and flows that involve bodies undergoing dynamic motion and deformation.

## Methods

### Force Partitioning Method (FPM)

The starting point for our formulation is the incompressible Navier-Stokes equation in the Lamb-Gromeka form, which is written as follows:

$$\begin{array}{c} \rho \frac{\partial \vec{u}}{\partial t}+\rho \vec{\omega }\times \vec{u}+\frac{1}{2}\rho \vec{▽}(\vec{u}\cdot \vec{u})=-\vec{▽}p-\mu \vec{▽}\times \vec{\omega }; \end{array}$$ (2)

where *ρ* and *μ* are the fluid density and absolute viscosity, and $\vec{u}$, *p* and $\vec{\omega }$ are the flow velocity, pressure and vorticity respectively. The above equation is augmented by the incompressibility constraint and the velocity boundary conditions as described in detail in S2 Text.

Following the approach of Quarterpelle & Napolitano [15] and Magnaudet [16], a harmonic function Φ^(*i*)^ for *i* = 1,2,3 is now introduced, which satisfies at any time-instance *t* = *τ*, the following equation:

$$\begin{array}{c} \nabla^{2}\Phi^{\left(i\right)}\left(\tau \right)=0\;\text{with}\;\;\hat{n}\cdot \vec{\nabla }\Phi^{\left(i\right)}=\{\begin{array}{cc} n_{i}, & \text{on}\;B(\tau )\\ 0, & \text{on}\;\Sigma \end{array} \end{array}$$ (3)

Given the above prescription, Φ^(*i*)^(*τ*) can be identified as the potential associated with the ideal flow past the body with shape and location corresponding to *t* = *τ*, translating in the *x*_*i*_ direction with constant unit velocity. Furthermore, this “unipotential” Φ^(*i*)^ for *i* = 1,2,3 can be obtained by solving Eq (3) individually for each of the three components. Eq (2) is now projected onto the space of $\vec{\nabla }\Phi^{\left(i\right)}$ and the resulting terms volume-integrated over *V*_*f*_. Subsequently by using Eq (3) and the divergence theorem to simplify the terms on the right-hand side of the equation, and by further applying the Helmholtz velocity decomposition (see S3 Text), we obtain the following final expressions or the partitioning of the total force on the wing:

$$\begin{array}{c} F_{B}^{i}=F_{\kappa }^{i}+F_{\omega }^{i}+F_{\sigma }^{i}+F_{\phi }^{i}+F_{\Sigma }^{i}\;\text{for}\;\;i=1,2,3;\;\text{where} \end{array}$$ (4a)

$$\begin{array}{c} F_{\kappa }^{i}=\underset{F_{\kappa_{\text{I}}}^{i}}{\underset{⎵}{-\rho \int_{B}\frac{d\vec{U}}{dt}\cdot \hat{n}\Phi^{\left(i\right)}dS}}\;\underset{F_{\kappa_{\text{II}}}^{i}=0}{\underset{⎵}{-\rho \int_{B}\frac{1}{2}U^{2}\hat{n}\cdot \vec{\nabla }\Phi^{\left(i\right)}dS}} \end{array}$$ (4b)

$$\begin{array}{c} F_{\omega }^{i}=\rho \int_{V_{f}}\vec{\nabla }\cdot [\left(\vec{\omega }\times \vec{u}\right)+\vec{\nabla }(\frac{1}{2}u_{v}^{2}+\left({\vec{u}}_{\phi }\cdot {\vec{u}}_{v}\right))]\Phi^{\left(i\right)}dV \end{array}$$ (4c)

$$\begin{array}{c} F_{\sigma }^{i}=-\mu \int_{B}(\vec{\omega }\times \hat{n})\cdot \vec{\nabla }x_{i}dS+\mu \int_{B}(\vec{\omega }\times \hat{n})\cdot \vec{\nabla }\Phi^{\left(i\right)}dS \end{array}$$ (4d)

$$\begin{array}{c} F_{\phi }^{i}=\rho \int_{V_{f}}\vec{\nabla }\cdot [\left(\vec{\nabla }\frac{1}{2}u_{\phi }^{2}\right)\Phi^{\left(i\right)}]dV\approx 0 \end{array}$$ (4e)

$$\begin{array}{c} F_{\Sigma }^{i}=-\rho \int_{\Sigma }\hat{n}\cdot \frac{d{\vec{v}}^{\prime }}{dt}\Phi^{\left(i\right)}dS+\mu \int_{\Sigma }(\vec{\omega }\times \hat{n})\cdot \vec{\nabla }\Phi^{\left(i\right)}dS\approx 0 \end{array}$$ (4f)

where $\vec{U}$ is the velocity of the surface of the wing and $\vec{u_{\phi }}$ and $\vec{u_{v}}$ are the curl-free (irrotational) and solenoidal (rotational) components of flow velocity, respectively [17].

In the above equations, *F*_*κ*_ is a force component that is determined solely by the kinematics (shape, velocity and acceleration) of the wing. In claiming this, we start by reemphasizing that as per Eq (3), the unipotential Φ only depends on the shape of the immersed body and is independent of the surrounding velocity field as well as the boundary conditions on the outer boundary. In the first integral in this term, *F*_*κ*_I__, $d\vec{U}/dt$ denotes the material acceleration of the immersed body and this force component is therefore totally independent of the flow velocity and vorticity in the surrounding fluid; in fact, this term represents the total force due to acceleration reaction. The second integral in *F*_*κ*_ (denoted by *F*_*κ*_II__) depends only on the surface velocity and Φ, and is also determined totally by the kinematics of the body.

Given that ${\vec{u}}_{v}=0$ if $\vec{\omega }=0$ (see details in S3 Text), the force component *F*_*ω*_ is identically zero if $\vec{\omega }$ is zero; this force component is therefore associated exclusively with the vorticity in the flow. *F*_*σ*_ consists of the viscous wall shear (first term in *F*_*σ*_) as well as pressure induced by the viscous dissipation in the flow (second term in *F*_*σ*_). *F*_*ϕ*_ is the force associated exclusively with the curl-free or potential component of the flow (${\vec{u}}_{\phi }$) within the domain. Finally, *F*_Σ_ is the force contribution associated with the flow perturbation ($\vec{v^{\prime }}$) and viscous shear on the outer boundary of the domain. *F*_*κ*_II__ is identically zero for zero-thickness membranes such as the wings modeled here, and as shown in S2 Text, *F*_*ϕ*_ and *F*_Σ_ are negligible compared to the other components for the cases simulated here; we therefore focus our attention on *F*_*κ*_I__, *F*_*ω*_ and *F*_*σ*_, forces respectively due to acceleration reaction, vorticity and viscosity.

It is noted here that given the non-linearity of the Navier-Stokes equations, the decomposition of the aerodynamic forces is not unique. In fact, decompositions based on a rigorous mathematical treatment of the Navier-Stokes include those of Wu [18], Noca et al.[19], Wu [20], Quartapelle and Napolitano [15], Howe [21], Magnaudet [16] and Wang & Eldredge [22]. The latter four references are particularly relevant for the current work since there is similarity, and perhaps, even some equivalence, in the methodology and/or force components resulting from these as well as the current decomposition. There have also been efforts to decompose the forces, especially those on a flapping wing, via a phenomenology-based approach; these include the studies of Ellington and coworkers [1, 3] who attempted to separate the effect of wing rotation versus linear flapping, and those of Dickinson and coworkers [7, 8] who focused on delineating the effect of wing rotation from that of quasi-steady aerodynamics. Depending on the objective of the researcher, a particular decomposition might be more appropriate than another to answer a specific question. For instance, the decomposition of Dickinson et al. [7] is effective in answering the oft-posed question as to how unsteady effects associated with flapping generate lift forces over and above those produced by equivalent quasi-steady aerodynamic mechanisms. However, this method does not for instance, separate the effects of vorticity from other mechanisms since both the rotational lift and the quasi-steady lift components (as defined by them) contain direct contributions from vorticity.

### Flapping Wing Models

Flapping kinematics derived from two insects, a hawkmoth and a fruit fly, form the basis of the current study (Fig 1c and 1f). Acquisition and processing of the hawkmoth flapping kinematics data was previously described in detail in Zheng et al. [23]. In brief, a male hawkmoth (*Manduca sexta*) was recorded hovering in front of an artificial flower via 3 calibrated, orthogonally positioned high speed video cameras (2x Phantom v7.1 and 1 Phantom v5.1, Vision Research, Wayne NJ) operating at 1000 frames per second. The 0.37 *m* ^3^ flight chamber was lit in the infra-red at 760 *nm* to allow camera operation without disturbing the animal. Body and overall wing kinematics were collected by marking discrete locations in the 3 stereo-calibrated views. Flexible wing mesh kinematics were collected by reconstructing the scene in the 3D animation package Maya (Autodesk Inc., San Rafael, CA) and hand-adjusting a multi degree of freedom wing mesh to match the wing shape observed in the images, a process similar to the scientific rotoscoping method sometimes used to produce 3D skeletal kinematics from stereo x-ray videography [24].

Fruit fly data were acquired for this study using two orthogonally positioned Phantom v7.1 high speed cameras (Vision Research, Wayne, NJ) equipped with 55mm Micro-Nikkor AIS lenses (Nikon Inc., Melville, NY) and 1.4x extension tubes operating at 4000 frames per second. The cameras were calibrated using a structure from motion approach employing sparse bundle adjustment [25] and a 3.43 mm calibration wand. The resulting calibrated imaging volume was a cube with sides of approximately 1.4 cm. A population of Canton-S *Drosophila melanogaster* was placed in a pipette tip pointing upward with the tip at the bottom of the imaging volume and allowed to take flight voluntarily. Flies were recorded shortly after takeoff, an individual which stabilized its body orientation within the imaging volume was selected for further analysis and three consecutive wingbeats were digitized at the following landmarks: tip of head, tip of abdomen, left and right wing base, left and right wing tip, left and right wing trailing edge at 2/3rds span. The 95% confidence interval width for these landmarks was 0.07 *mm* in each direction. The perimeter of the left wing was digitized in a single video frame for calculation of wing area and wing length, 2.58 *mm* ^2^ and 2.72 *mm* respectively. Kinematic analysis of overall body motion via the tip of head and tip of abdomen points revealed that the fly was moving upward at 0.41 *m* *s* ^−1^ (∼ 0.7 body lengths per flapping cycle) and accelerating upward at 0.74 *m* *s* ^−2^. The body length of the fly was 2.67 *mm*; following [26] we modeled the body as a cylinder with radius 15% of the length, and a density 1.039 *kg* *m* ^−3^ resulting in an estimated body mass of 1.40 *mg*. Together with the upward acceleration against gravity but ignoring drag, this indicated that the fly produced an upward flight force of 1.48 × 10^−5^ *N*.

The three successive wingbeats were averaged together and the rigid wing motion angles extracted as shown in Fig 1f, producing the kinematics shown in Fig 1d and 1f. The flapping frequency was found to be 222±3.8 (mean±s.d.) Hz. Note that these kinematics recorded shortly after takeoff differ from both the idealized hovering kinematics used in earlier physical and computational models of fly flight [7, 12, 27] and also from kinematics recorded in free flying flies long after takeoff [28]. In particular, the fly recorded here exhibited a greater downward tilt to its overall stroke plane;58.7±2.5° as compared to 1.3° in the well resolved hovering kinematics presented in [28]. Other aspects of the stroke were more similar; we found an overall flapping amplitude of 141±2.9° and wing pitching amplitude of 113±1.4° compared to 146° and 132° in [28], respectively. Despite these differences, the kinematics are representative of those employed by a fly supporting its weight in the air (see below) and are thus a useful point of comparison for the hawkmoth data. Furthermore, recent work in flies [29, 30] has revealed a wide variety of flapping kinematics beyond “normal” hovering, and the Navier-Stokes simulation of the alternative kinematics here provides additional data on the time course of force production during the flapping cycle of the fly.

The nominal wing Reynolds number, defined as $Re={\bar{V}}_{\text{tip}}\bar{c}/\nu$, where ${\bar{V}}_{\text{tip}}$ and $\bar{c}$ are the average wingtip velocity and span-averaged wing-chord respectively, were chosen to be 1000 for the hawkmoth and 100 for the fruit fly. In additional to these nominal cases, a wider range of Reynolds numbers were also explored in the latter part of the study.

### Flow Solver

Simulations for these and other cases discussed on this paper are carried out with an immersed boundary flow solver [14]. The flow solver models incompressible, viscous, time-dependent flows over complex stationary and moving boundaries on stationary Cartesian grids with a sharp-interface immersed boundary method that is second-order accurate in both space and time. The grid topology and the computational domain employed in these simulations is shown in Fig 1a and 1b. Based on our previous experience in simulating such flows [9], grids with sizes ranging from 10 to 16 million points were chosen for the current simulations, and time-steps per flapping cycle ranged from 2500 to 4200. With these grids, it has been shown [9] that both the time-averaged as well as the time-varying (root-mean-square) component of the aerodynamic force on the insect wings has a less than 1% variability with the grid resolution.

The flow solver was augmented to perform the FPM analysis concurrently with the simulation. This requires the solution of the Laplace Eq (3) for the unipotential Φ at each time step. Subsequently, each term in Eq (4) was computed at each time-step by combining the velocity and vorticity field obtained from the flow simulation with the computed Φ. Further details of the methodology and assessment of truncation errors in the FPM analysis can be found in S2 Text.

## Results and Discussion

**Fig 2:**
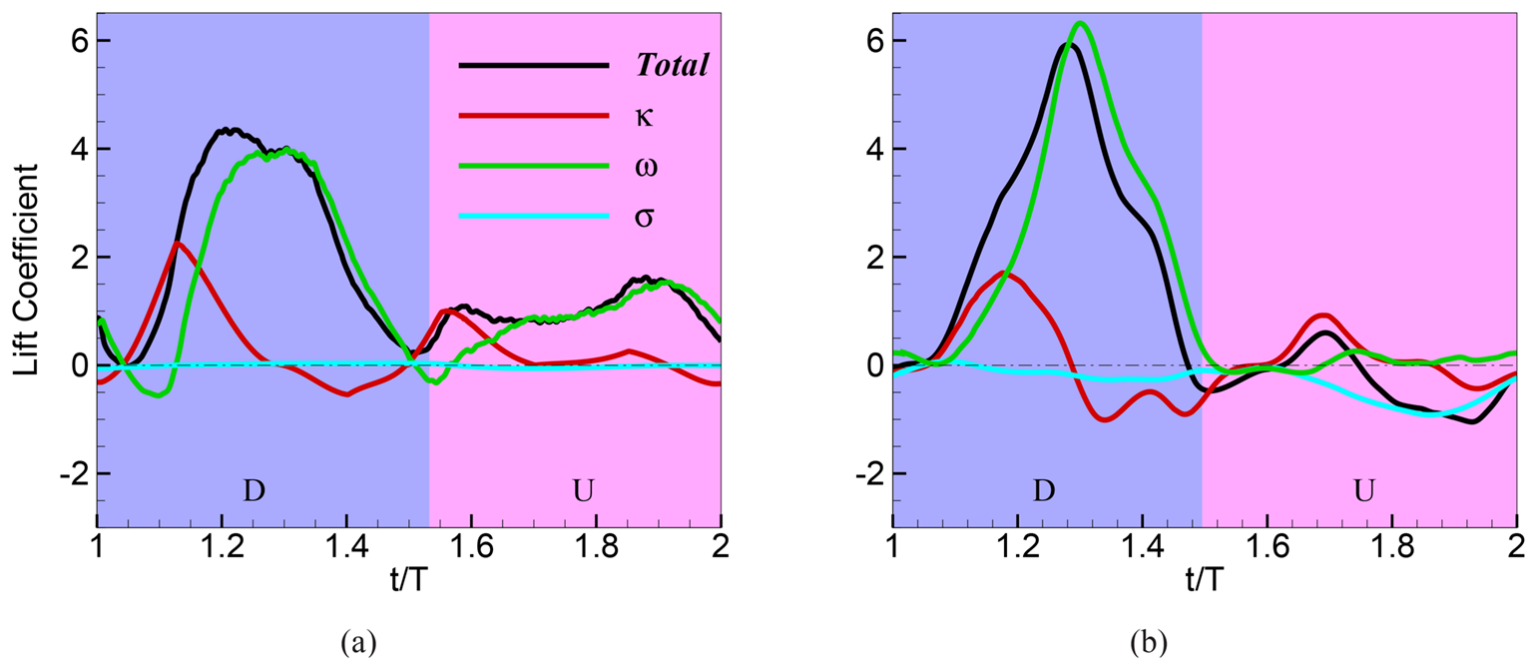
Components of the instantaneous lift coefficient over one flapping cycle for the (a) hovering hawkmoth at Re = 1000, and the (b) fruit fly at Re = 100. The stroke is divided into two phases: downstroke (D) and upstroke (U). Lift coefficient is defined as *C*_*F*_ = *F*/(*ρAβ* ^2^ *f* ^2^ *L* ^2^) where *F* is force, *ρ* is the fluid density, *A* and *L* the wing area and wing length respectively, and *β* and *f* the stroke amplitude and frequency respectively. The vortex-induced lift (VIL) *F*_*ω*_ exhibits a large and distinct peak near mid-downstroke for both wings. In upstroke both wings generate positive VIL, but the magnitudes are significant only for the hawkmoth.

Fig 2 shows the time variations of the total lift coefficient (which corresponds to the vertical direction *x*_2_) and its three significant components (*F*_*κ*_I__, *F*_*ω*_, and *F*_*σ*_) for the two cases studied here. Table 1 summarizes the time-averaged values of these components over one flapping cycle. For the hawkmoth wing, the downstroke contributes the majority (70%) of the total lift and the upstroke, 30%. As described in detail in our previous paper [9], the simulations of the hovering hawkmoth reproduce both the mean as well as time-variation of the lift quite accurately when compared to the experimental estimates obtained from the body acceleration of the animal during flight. In contrast to hovering flight of the hawkmoth, the fruit fly in slow climbing flight modeled here, generates all of its weight support during the downstroke, with the upstroke and wing rotation periods generating a small net *negative* lift force. Thus, compared to the idealized hovering kinematics [7] or kinematics measured from freely hovering flies [11], which produce similar magnitude lift in upstroke and downstroke and, depending on rotation phase, substantial forces during wing rotation, the tilted stroke plane kinematics adopted by the fly recorded here differ markedly in the time course of force production within a stroke. However, when re-dimensionalized for comparison with the whole-body kinematics of the fruit fly (described above), our simulation predicts a net upward force of 1.83 × 10^−5^ *N*, or 124% of the 1.48 × 10^−5^ *N* calculated from whole-body kinematics and body mass. Note that our whole-body kinematics does not include the drag due to upward motion, so the computed 1.48 × 10^−5^ *N* upward force is expected to underestimate the force actually produced by the fly. Thus, although the flapping kinematics used by the fly here are distinct from typical hovering, they also produce force sufficient to support the weight of the fly and even account for its slight upward acceleration. It is not clear why the fly used wing kinematics distinct from those typically recorded from hovering animals, but it may be the case that tilting the stroke plane down and concentrating force production at the phase of the stroke cycle when the wing is moving downward improves efficiency during climbing flight. The figure and the table indicate that the viscosity-induced lift force is nearly negligible for the hawkmoth and it generates a non-negligible (25%) but net-negative lift contribution for the fruit fly. This is consistent with the order-of-magnitude higher Reynolds number for the hawkmoth. The rest of the discussion will focus on the vortex-induced and kinematic lift components.

**Table 1:** Stroke-averaged lift coefficients $\bar{C_{F}}$ for the hawkmoth (Re = 1000) and fruit fly (Re = 100) wings. Values in parentheses denote the percentage of the total attributed to the given component. Overall, the vorticity in the flow contributes 82.5% of the total lift for the hawkmoth and ∼ 114% of the net lift over one stroke of the fly wing. This greater than 100% contribution results from the fruit fly’s operation at a lower Reynolds number, where viscous force induces a sizeable (25% of the net) negative lift over the cycle. The balance of the positive lift for both insects comes from the kinematic component *F*_*κ*_; 17.3% for the hawkmoth and 9.5% for the fruit fly.

	*F*_*κ*_+*F*_*ω*_+*F*_*σ*_		*F*_*κ*_		*F*_*ω*_		*F*_*σ*_
	$\bar{C_{F}}$	$\bar{C_{F}}$	% of total	$\bar{C_{F}}$	% of total	$\bar{C_{F}}$	% of total
Hawkmoth	1.65	0.29	17.3%	1.36	82.5%	0.00	0.2%
Fruit fly	1.06	0.10	9.5%	1.22	114.5%	-0.27	-25.0%

### Vortex-Induced Lift and LEV Contribution

**Fig 3:**
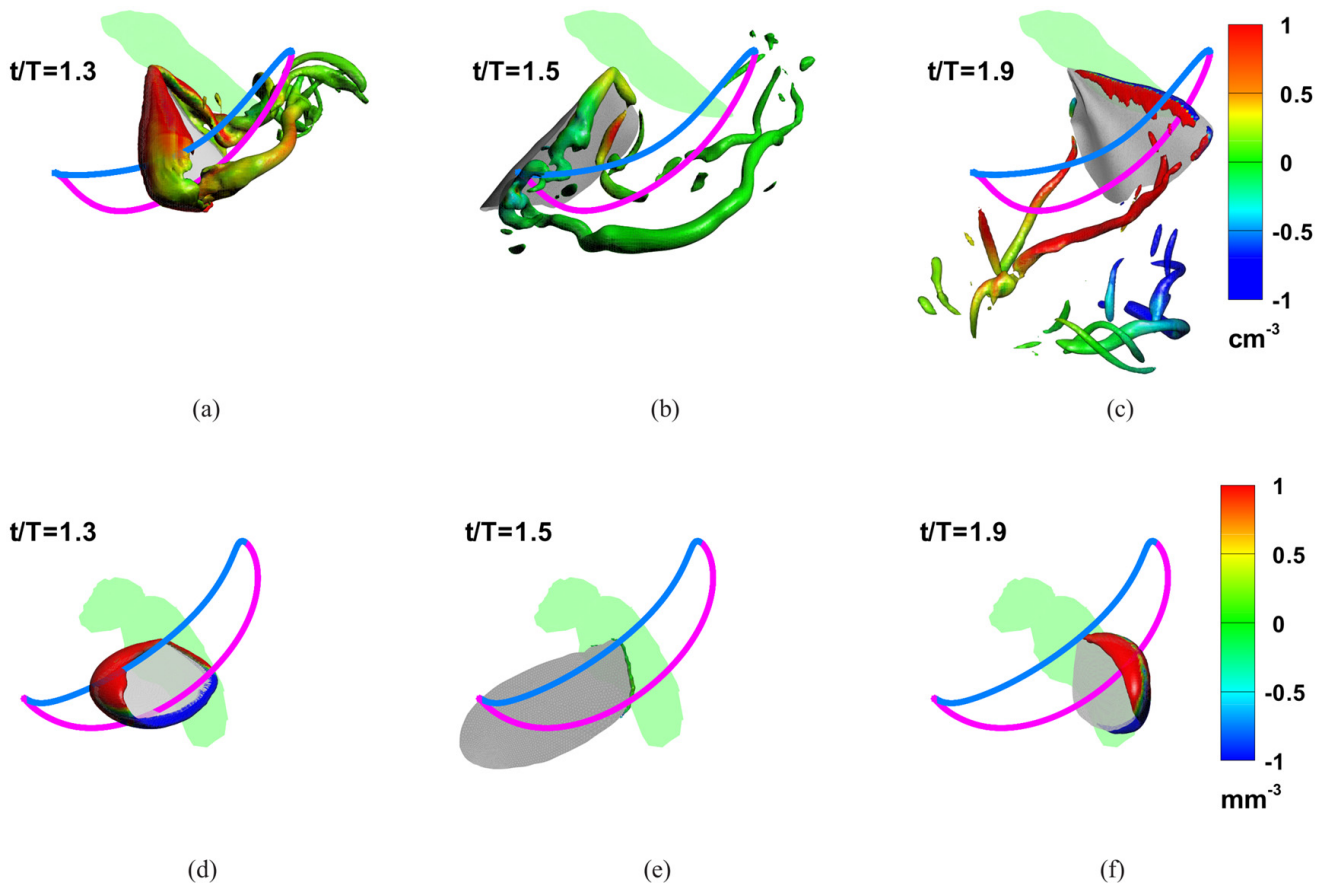
Vortex structures and local contribution of vortices to lift production. Vortex structures are identified by plotting an isosurface of the imaginary part of the complex eigenvalue of the local deformation tensor [31]. Isosurfaces are shaded by the lift coefficient per unit volume (*cm* ^3^ for the hawkmoth and *mm* ^3^ for the fruit fly) contributed by local vorticity. Panels (a-c) and (d-e) show the three phases in the flapping of the hawkmoth and fruit fly respectively. The hawkmoth wing, which is operating at a higher Reynolds number, generates a number of distinct vortices including the LEV, a tip-vortex and a root-vortex. (a) the downstroke peak in VIL for the hawkmoth wing, the bulk of the lift-producing vorticity patches are associated with the LEV. (b) at *t*/*T* = 1.5, the end downstroke, there is a spiralling LEV and a strong tip-vortex but neither of them generates much lift. (c) at *t*/*T* = 1.9, at the peak in VIL during late upstroke, the LEV on the ventral surface of the wing is weak but the wing-tip vortex provides a noticeable contribution to lift. For the fruit fly wing, which operates at a lower Reynolds number, the LEV is *the* most dominant vortical structure in downstroke (d) and upstroke (f); vorticity at the end of downstroke (e) is negligible.

Fig 3 shows the vortices at three key stages in the flapping cycles for the two insects. The vortex structures are identified by plotting an iso-surface of swirl-strength; swirl strength is the imaginary part of the complex eigenvalue of the local deformation tensor [31], which is a well-accepted, frame-invariant quantity for identifying vortices. For the hawkmoth, we note the formation of a leading-edge vortex at mid-downstroke (*t*/*T* = 1.3); this vortex is conical in shape as noted by Ellington and coworkers [2, 3] as well in the computational studies of Liu and coworkers [32, 33], and it connects with the tip-vortex as well as a weak proximal-edge vortex. Near the end of the downstroke (*t*/*T* = 1.3), the LEV shows the spiral topology that has also been described before in experiments [2, 3] as well as simulations [32] and is a characteristic feature for flapping wings at O(1000) Reynolds number [5]. The tip-vortex in the current simulations is also observed to extend a significant distance from the wing-tip. The vortex structure at end-upstroke (*t*/*T* = 1.3) is dominated by tip vortices from the proximal and distal wingtips; also noticeable at this phase is a much smaller LEV on the ventral surface of the wing, as well as the remnants of the wake vortices from the downstroke that are located about one wing-span below the wing.

Several studies [2–5, 32] have noted the importance of the leading-edge vortex in flapping wing flight, few have attempted to estimate the actual contribution of the LEV to total lift. Ellington et al. [2] and Vanderberg et al [3] attempted to estimate the contribution of the LEV for their mechanical hawkmoth flapper using smoke visualization, and suggested that the LEV could provide up to 2/3^rd^ of the total lift needed for flight. Given the highly three-dimensional and transient nature of the LEV and the limitations of smoke visualization in providing quantitative flow information, the uncertainty in the above estimate is difficult to quantify. Bomphrey et al. [34] also attempted to estimate the lift associated with the LEV of flapping insect wings; they used digital particle-image velocimetry (DPIV) to measure flow around hawkmoths tethered in a uniform flow with wind speeds of 1.2 and 3.5 *m* *s* ^−1^ (∼ 1.3 and 3.7 body lengths per flapping cycle given *f* = 19 *Hz* and assuming a body length of 5 *cm*) and estimated the circulation of the LEV. By applying the Kutta-Joukowski theorem [35], to the vortex on the midwing plane, they estimated that the LEV could provide about 13% of weight support at the lower speed and about 64% of weight support at the faster speed. This result is surprising since at the lower speed, more of the weight support should come from unsteady mechanisms such as the LEV because the contribution of steady mechanisms are expected to be proportional to the square of the freestream velocity and therefore much less at the lower speed. It is not clear if the tethering (the body of the animal was glued to a sting) produced a biologically unrealistic response in the animal, or if the lift estimates are affected by other assumptions and technical limitations inherent in this early application of DPIV to insect flight.

Since the integral in *F*_*ω*_ in Eq (4) can be evaluated for every local patch of vorticity in the computed field, the FPM can be deployed to determine the contribution of the LEV and other distinct vortices to lift with a precision that has not been available before. Fig 3 shows the vortices over the wings color-coded for their contribution (per unit-volume) to *F*_*ω*_. By limiting the volume of integration to the region occupied by the LEV as identified by the swirl strength, we find that for the hawkmoth wing, the LEV contributes about 85% percent of the total VIL at mid-downstroke (Fig 3a), with the balance of the VIL at this phase coming mostly from the tip-vortex and the attached proximal-edge vortex. During late upstroke, the peak in the vortex lift occurs at around *t*/*T* = 1.9 and this peak is associated with the weak LEV on the ventral surface of the wing, as well as the two tip vortices that come off both the distal and proximal edges of the wing. It is also noted that the wake vortices from the downstroke that are located below the wing actually make a small but negative contribution to the lift at this stage. Thus “wake-capture” [7] in this case, although not very significant, is actually detrimental to lift production.

By conducting a similar analysis of the LEV for a number of selected instances during the flapping stroke, and time-integrating the results, we find that the LEV contributes 87% of the VIL for the entire stroke and 72% of the total lift over one stroke. This value is fairly consistent with the estimate of Vanderberg & Ellington [3] that was in the 67% range for LEV lift contribution to weight support. The number predicted here is however much larger than the 13% figure for the lower freestream velocity case of [34], which would be considered closer to the current case of hover. As noted earlier, the trend for the slow and fast flight estimates for LEV lift in [34] are unusual and we believe that this further discrepancy with our results relates to the complexity inherent in obtaining such estimates in experiments, especially in those involving live animals.

As noted in Fig 3, the LEV is an even more dominant feature for the fruit fly during both the downstroke (Fig 3d) and upstroke (Fig 3e). The higher viscosity of this case dissipates all the other vortices (tip and wake vortices) so rapidly that they are difficult to detect in the flow. There is also no evidence of any spiraling motion in the core of the LEV, consistent with the observation of Birch et al. [5] and [32] for flapping wings at O(100) Reynolds numbers. During early downstroke (Fig 3d) the wing also generates an attached vortex on the *trailing-edge* of the wing which provides a negative lift contribution. Similarly, during late upstroke (Fig 3f), there is an attached vortex on dorsal side of the wing-tip that produces a negative lift contribution. Not surprisingly, the LEV produces almost all (98%) of the VIL.

### Added-Mass and Centripetal Acceleration Reaction

In addition to quantifying the role of the various vortices on lift production, the current analysis reveals that the total added-mass force *F*_*κ*_I__ also generates a sizeable net positive contribution to lift for both wings. For the hawkmoth, *F*_*κ*_I__ contributes about 17% to the total net lift over one stroke whereas for the fruit fly, *F*_*κ*_I__ contributes about 10% to the net lift. The physical origin of the force associated with *F*_*κ*_I__ is revealed by noting that the surface velocity can be decomposed as $\vec{U}={\vec{U}}_{\phi }+{\vec{U}}_{v}$, where, as shown in S3 Text, ${\vec{U}}_{\phi }$ and ${\vec{U}}_{v}$ are the flow velocities on the surface associated with the potential and viscous flow components respectively. *F*_*κ*_I__ can therefore be decomposed as follows:

$$\begin{array}{c} F_{\kappa_{\text{I}}}^{\left(i\right)}=-\rho \int_{B}\frac{d\vec{U}}{dt}\cdot \hat{n}\Phi^{\left(i\right)}dS=\underset{F_{\kappa_{i}}^{\left(i\right)}}{\underset{⎵}{-\rho \int_{B}\frac{d{\vec{U}}_{\phi }}{dt}\cdot \hat{n}\Phi^{\left(i\right)}dS}}\;\underset{F_{\kappa_{v}}^{\left(i\right)}}{\underset{⎵}{-\rho \int_{B}\frac{d{\vec{U}}_{v}}{dt}\cdot \hat{n}\Phi^{\left(i\right)}dS}}; \end{array}$$ (5)

**Fig 4:**
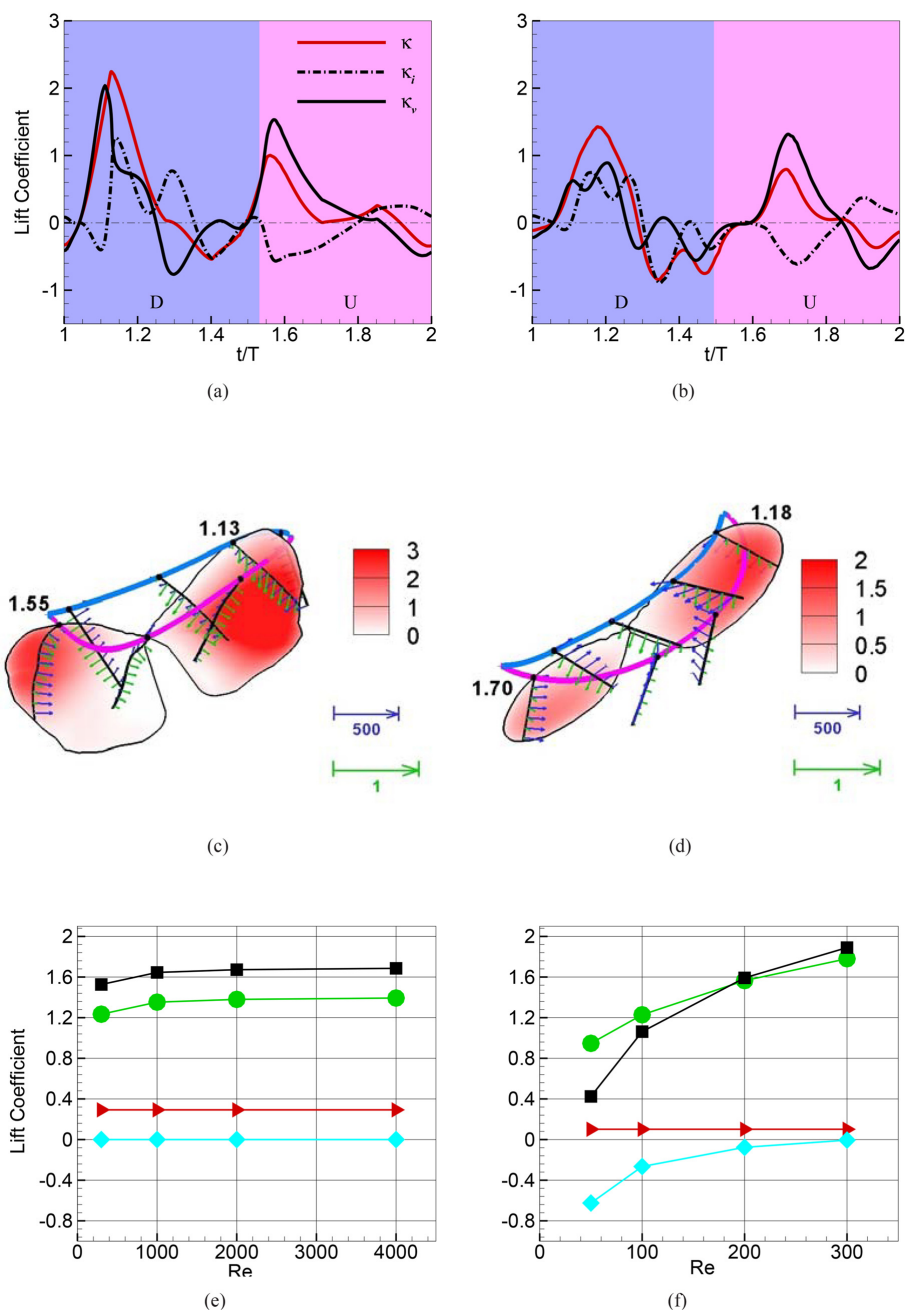
Time variation of the components of added-mass lift $F_{\kappa_{\text{I}}}^{\left(2\right)}$ for the (a) hawkmoth and (b) fruit fly over one flapping cycle. Surface contours of added-mass lift $F_{\kappa_{\text{I}}}^{\left(2\right)}$ at instants corresponding to peak values in the flapping cycle for the (c) hawkmoth and (d) fruit fly. Chord-lines at a few instances with black and green vectors corresponding to $d\vec{U}/dt$ and $-\hat{n}\left(\Delta \Phi^{\left(2\right)}\right)$ respectively where ΔΦ^(2)^ is the difference in scalar Φ^(2)^ on the two sides of the wing, are also shown. Note the supination and downward movement of the wing in early upstroke. During this time, the vectors corresponding to $d\vec{U}/dt$ and $-\hat{n}\left(\Delta \Phi^{\text{(2)}}\right)$ are pointing in similar directions so that their dot-product generates a positive (upward) lift force, creating a net positive centripetal acceleration reaction contribution during upstroke. Scaling of various components of lift with Reynolds numbers for the (e) hovering hawkmoth and (f) fruit fly. The black, green, red and cyan lines represent total, vortex-induced, added-mass and viscous components of lift, respectively.

As shown in S4 Text, the first term $F_{\kappa_{i}}^{\left(i\right)}$ on the right hand side of Eq (5) is the classical inviscid added-mass force [36, 37] that is experienced by a body accelerating in a potential flow. However, it is well established [38] that the inviscid added-mass cannot generate a net force on a body such as a flapping wing that is undergoing a periodic motion (see S4 Text). Consistent with this, the stroke-averaged value of the inviscid added-mass force $F_{\kappa_{i}}^{\left(i\right)}$ is indeed found to be negligible (Fig 4a and 4b) for both flapping wings studied here. It is noted that this classical added-mass force has been examined in detail by Kang et al. [39] for flexible flapping wings and their expression for the added-mass of such wings (Eqs 5.9 in their paper) also shows a net zero added-mass force contribution to lift. Perhaps the most convincing demonstration that the classical inviscid added-mass generates zero net lift for a periodically flapping wing is by Whitney and Wood [40] who conducted a comprehensive experimental study of a flexible flapping wing. The force due to the classical added-mass was calculated by applying the theory of Sedov [36] to the measured motion of the wing, and the net contribution of the added-mass to lift is nearly zero (see Fig 4a and 4b of the paper). It should be noted that despite integrating to zero over a periodic flapping cycle, added-mass forces are not necessarily unimportant for insect flight. This is because the instantaneous values of the added-mass force during the flapping cycle may be non-negligible (in fact, in the current study, the peak value of the added-mass lift is nearly one-fourth of the peak total lift for the hawkmoth and about one-sixth for the fruitfly) and instantaneous added mass forces could therefore lead to effects such as wing deformation, which could subsequently alter the mean lift generated by the other mechanisms.

From the above discussion, it therefore follows that it is the second term *F*_*κ*_*v*__ in *F*_*κ*_I__ that generates the non-zero net value for this force component. We note that for the second term to be non-zero the following two conditions need to be satisfied: first, the flow needs to satisfy the no-slip condition; second, the tangential acceleration of the surface should have a non-zero component along the surface normal direction, and this second condition requires rotation of the body with a center located away from the local surface. The term in Eq (5) is therefore associated with the surface pressure needed to generate the centripetal acceleration of the fluid layer that is immediately adjacent to the surface, and can be thought of as a “centripetal” acceleration reaction force. In contrast, the classical added-mass force is a linear acceleration reaction, which is associated with the movement of the body relative to the fluid in a direction normal to the body surface, and it has no association with the no-slip condition. As shown in Fig 4c and 4d, the deformation and rotation of the hawkmoth wing, especially of the leading-edge in early downstroke, explains the greater contribution of centripetal acceleration reaction to lift in the hawkmoth versus the rigid wing fruit fly cases.

Thus, our analysis shows that while the LEV contributes most of the net lift for these hovering insects, there is another mechanism, the centripetal acceleration reaction, that also contributes to net lift. This mechanism has a number of distinguishing features. First, it is similar to the classical inviscid added-mass force that is a reaction to the acceleration of the body, but is different in that: (a) it requires the satisfaction of the no-slip condition; and (b) it is not linear acceleration of the body in a direction normal it its surface but rather, centripetal acceleration of the layer of fluid adjacent to the surface that generated this force. While acceleration reaction forces have received some attention in the context of biological locomotion, e.g. by Daniel [41] and viscous acceleration reaction forces are not unknown in fluid dynamics (consider the Basset force [42] produced by the acceleration associated lag in the development of the boundary layer), to our knowledge, the viscous centripetal acceleration force generation mechanism described here, has neither been identified before in fluid dynamics nor in the study of flapping flight.

A second interesting feature of this lift component is that the two largest positive peaks in *F*_*κ*_I__ for the insects coincide with phases in the stroke where the vortex-induced lift is small or even negative. Thus, in addition to contributing to overall weight support, the centripetal acceleration reaction would also reduce the time-variation in the total lift. A third distinguishing feature of the centripetal acceleration reaction is that while it is dependent on the satisfaction of the no-slip condition on the flapping wing, which necessitates a non-zero viscosity, the mathematical expression for this force is independent of the actual magnitude of viscosity. This is because this force is associated with the infinitesimally thin bound-vortex sheet on the body created by the no-slip condition and the thickness of this sheet is independent of the Reynolds number. Thus, for the same wing shape and wing kinematics, a change in Reynolds number has no effect on the lift coefficient associated with the centripetal acceleration reaction; Fig 4e and 4f show that the vortex-induced lift decreases with Reynolds number for both cases, whereas the lift due to the centripetal acceleration reaction *F*_*κ*_I__, remains constant. At Re = 50, the centripetal acceleration reaction lift is 25% of the total for the fruit-fly and this suggests that centripetal acceleration reaction lift could be increasingly important for flight at very small scales, such as for insects like thrips and parasitoid wasps.

Fourth, given that the centripetal acceleration reaction depends only on the surface motion, fluid density and the potential Φ (which also only depends on the geometry and orientation of the wing), this force is wholly independent of the surrounding flow and vorticity, in as much as the kinematics of the wing are not affected by these disturbances. Thus, insects flying in turbulent or otherwise disturbed airflows can rely on this mechanism of lift regardless of the environmental conditions as long as the kinematics of the wing are not affected.

It is worthwhile to examine the above results within the context of the possible numerical and modeling uncertainties. Numerical uncertainties are relatively easy to evaluate and as mentioned earlier, these are quite small. Modeling errors are more difficult to assess. In our previous paper [9], we described a very detailed validation of the computed lift for the hovering hawkmoth against that measured in the associated experiments. The comparison indicated that the computed time-varying lift matched very well with the experiment, even if the uncertainty in the experiments was discounted. Thus, the modeling errors for the hawkmoth simulations seem to be quite small. For the fruitfly, we do not have equivalent experimental data for direct comparison but since we use the same computational procedure and grid/domain as that of the hawkmoth, it is expected the flow physics and forces are correctly capture for the given wing geometry and kinematics. To what degree and generality does our flapping wing model represent fruitflies, is however difficult to assess. It is for this very reason that two very different animals that have very distinct flapping regimes/wings/kinematics have been modeled, and also one of the reasons why simulations have been conducted for a range of Reynolds numbers for each case. By compiling the results from all these simulations, it can be concludes that lift due to centripetal acceleration reaction may contribute in the range of 10–20% to the net lift for flying insects.

## Summary

In summary, the force-partitioning methodology (FPM) leads to a mathematically unambiguous and physically insightful partitioning of the fluid-dynamic forces experienced by a moving and/or deforming body immersed in a fluid. Using this method, we have precisely quantified the contribution of the leading-edge vortex to lift production for two flying insects, the hawkmoth and the fruit fly. We have also identified a new mechanism, the centripetal acceleration reaction, that contributes significantly to the total lift. This mechanism, which depends solely on the wing kinematics, is independent of the Reynolds number and environmental flow disturbances. It therefore provides an effective and robust mechanism for weight support at all scales.

While the focus of this paper is on insect flight, the FPM has wide ranging applications to virtually all fields of fluid dynamics, and in particular, to vortex dominated flows and flows with dynamically moving bodies. Similarly, the mechanism of centripetal added-mass force that has been identified here likely plays an important role in flows that involve bodies undergoing complex motions such as those encountered in the flying and swimming of animals, flow-induced vibration and deformation in biology and engineering, and multiphase flows.

## Supporting Information

S1 Dataset Data files corresponding to data in Figs 1e, 1f, 4e and 4f. (ZIP)

S1 Fig Schematics of control volume and internal body. (EPS)

S2 Fig Comparison of lift calculation by the traditional method (blue line) and the Force Partitioning Method (FPM) (black line) in (a) Hovering Hawkmoth flight at Re = 1000 and (b) Fruit fly at Re = 100 throughout a full stroke cycle. (EPS)

S1 Text List of Symbols. (PDF)

S2 Text Derivation of the Force Partitioning Method (FPM). (PDF)

S3 Text Helmholtz Decomposition. (PDF)

S4 Text Force on a Flapping Wing in Potential Flow. (PDF)

S5 Text Supporting Information Reference. (PDF)
