# Supplementary material for: Centripetal Acceleration Reaction: An Effective and Robust Mechanism for Flapping Flight in Insects
Source: PLoS One. 2015 Aug 7;10(8):e0132093. doi: 10.1371/journal.pone.0132093 (PMC4529139; doi:10.1371/journal.pone.0132093)
Supplement: S1 Text — (PDF) [file pone.0132093.s004.pdf]

# S1 List of Symbols

| | |
|---|---|
| $\bar{c}$ | Span-averaged wing chord |
| $\bar{V}_{\text{tip}}$ | Averaged wing tip velocity |
| $\beta$ | Stroke amplitude |
| $\hat{n}$ | Unit normal vector pointing outwards from the control volume |
| $\hat{t}$ | Unit tangential vector on the body surface |
| $\mu$ | Dynamic viscosity of fluid |
| $\overline{C_F}$ | Stroke-averaged force coefficient |
| $\Phi^{(i)}$ | Potential function for flow associated with body translating in $i$ direction with unit velocity. |
| $\rho$ | Density of fluid |
| $\Sigma$ | Outer boundary of control volume |
| $\vec{\omega}$ | Vorticity vector for the flow |
| $\vec{\tau}^{w}$ | Shear stress on the internal surface |
| $\vec{U}$ | Surface velocity of the internal body |
| $\vec{u}$ | Velocity vector of fluid |
| $\vec{U}_{\phi}$ | Surface flow velocity induced by potential flow |
| $\vec{u}_{\phi}$ | Velocity corresponding to Potential flow |
| $\vec{U}_{v}$ | Surface flow velocity associated with viscous part of flow obtained from Helmholtz Decomposition |
| $\vec{u}_{v}$ | Vortex induced velocity vector |
| $\vec{v}'$ | Perturbation in velocity at outer boundary due to flapping wing |
| $A$ | Wing area |
| $B$ | Internal body surface |
| $C_F$ | Force coefficient |
| $F$ | Force |
| $f$ | Flapping frequency |
| $F_{\kappa i}$ | Classical inviscid added-mass force |
| $F_{\kappa v}$ | Viscous centripetal acceleration reaction force |
| $F_{\kappa}$ | Total kinematic force |
| $F_{\omega}$ | Vortex induced force |

| | |
|---|---|
| $F_\phi$ | Inviscid force |
| $F_\Sigma$ | Outer boundary force |
| $F_\sigma$ | Viscous force |
| $F_B$ | Total force acting on internal body in i direction |
| $i$ | Direction; 1 for $x_1$, 2 for $x_2$ and 3 for $x_3$ |
| $L$ | Wing length |
| $n_i$ | Dot product of normal vector of surface and unit vector in $i$ direction |
| $p$ | Pressure |
| $r$ | Distance from the internal body |
| $Re$ | Reynolds number |
| $V_f$ | Control volume |
| $x_i$ | Local coordinate in $i$ direction |
| $U_{\phi_t}$ | Slip velocity associated with potential flow |
