# Supplementary material for: Centripetal Acceleration Reaction: An Effective and Robust Mechanism for Flapping Flight in Insects
Source: PLoS One. 2015 Aug 7;10(8):e0132093. doi: 10.1371/journal.pone.0132093 (PMC4529139; doi:10.1371/journal.pone.0132093)
Supplement: S2 Text — (PDF) [file pone.0132093.s005.pdf]

S2 Derivation of the Force Partitioning Method (FPM)

The partitioning of the forces experienced by a body immersed in a fluid into components that can be attributed to various physical mechanisms has been a recurrent theme in fluid dynamics and a variety of formulations have been developed that attempt to accomplish this. Among these, the derivative-moment transformation of Wu [1, 2] and the force projection methods of Quarterpelle & Napolitano [3], Howe [4], and the "reciprocal theorem" of [5] are particularly worth mentioning here. In the current work, we follow the force decomposition approach of Quarterpelle & Napolitano [3] but extend it in two ways: first by employing the Helmholtz velocity decomposition, we provide a clearer separation of the vortical components of force from the other components, and also separate the added-mass force into its inviscid and viscous components. Second, while Quarterpelle & Napolitano [3] and Magnaudet [5] limited the application of their formulations to canonical flows with relatively simple immersed bodies, we implement our force partitioning method (FPM) into a general flow-solver that enables the force partitioning to be applied to flows at finite Reynolds numbers with complex moving boundaries. The result is a powerful flow analysis tool that can be used to gain insights into the mechanisms of force production for a wide class of problems.

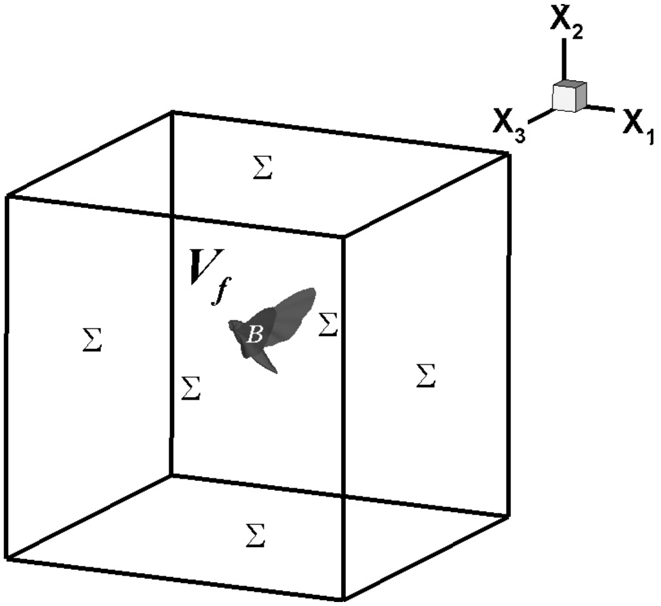

Figure S1. Schematics of control volume and internal body

We start with the incompressible Navier-Stokes equation in the Lamb-Gromeka form, which is written as follows:

$$\rho\frac{\partial\vec{u}}{\partial t}+\rho\vec{\omega}\times\vec{u}+\frac{1}{2}\rho\vec{\nabla}(\vec{u}\cdot\vec{u}) = -\vec{\nabla}p-\mu\vec{\nabla}\times\vec{\omega}; \tag{S1}$$

where ρ and μ are the fluid density and absolute viscosity, and $\vec{u}$, p and $\vec{\omega}$ are the flow velocity, pressure and vorticity respectively. The above equation is augmented by the incompressibility constraint and the boundary conditions (see schematic in S1 Fig.) for the above equation are

$$\vec{u}(\vec{x},t) = \begin{cases}\vec{U}(\vec{x},t), & \text{on } B(t)\\ \vec{V}_\infty+\vec{v}'(\vec{x},t), & \text{on } \Sigma\end{cases} \tag{S2}$$

that correspond to the flapping flight of an animal in a freestream with velocity equal to $\vec{V}_\infty$. In the above, $B(t)$ represents the time-dependent surface of the wing (and body), which may be moving and/or deforming, and $\vec{U}(t)$

is the velocity of this surface. Σ represents the outer surface of the large, but finite domain (with volume denoted by V_f) and $\vec{v}'$ represents the perturbation to the freestream on the outer boundary due to the immersed object. It is understood that for a large domain, $\vec{v}'$ would be much smaller than U. Our study here addresses hovering flight where $\vec{V}_\infty \equiv 0$. A schematic of the configuration is shown in S1 Fig. We note here that while the above conditions on the outer boundary are appropriate for hovering flight of an insect in a quiescent medium, the FPM method described here can also easily examine flows, both confined as well as unconfined, with more general boundary conditions.

A harmonic function $\Phi^{(i)}$ for $i = 1,2,3$ is now introduced, which satisfies at any time-instance $t = \tau$, the following equation:

$$\nabla^2 \Phi^{(i)}(\tau) = 0 \quad \text{with} \quad \hat{n} \cdot \vec{\nabla}\Phi^{(i)} = \begin{cases} n_i, & \text{on } B(\tau) \\ 0, & \text{on } \Sigma \end{cases} \tag{S3}$$

Given the above prescription, $\Phi^{(i)}(\tau)$ can be identified as the potential associated with the ideal flow past the body with shape and location corresponding to $t = \tau$, translating in the x_i direction with constant unit velocity. This "unipotential" $\Phi^{(i)}$ for $i = 1,2,3$ can be obtained by solving Eq. (3) in the main body individually for each of the three components. Eq. (S1) is now projected onto the space of $\vec{\nabla}\Phi^{(i)}$ and the resulting terms volume-integrated over V_f. Subsequently by using Eq. (S3) and the divergence theorem to simplify the terms on the right-hand side of the equation, we get

$$\rho \int_{V_f} \left(\frac{\partial \vec{u}}{\partial t} + \vec{\omega} \times \vec{u} \right) \cdot \vec{\nabla}\Phi^{(i)} dV = -\int_B \left(\frac{1}{2}\rho U^2 + p \right) n_i dS + \mu \int_{B+\Sigma} (\vec{\omega} \times \hat{n}) \cdot \vec{\nabla}\Phi^{(i)} dS \tag{S4}$$

We note in Eq. (S4) that due to the boundary condition in (S3), the integral corresponding to the pressure and kinetic energy at the outer boundary vanishes and thus, the pressure at the outer boundary, which is generally unknown, is factored out from the analysis, irrespective of the domain size.

The component of total force exerted on the body in i-direction can be expressed as,

$$F_B^i = \int_B p n_i dS + \int_B \vec{\tau} \cdot \vec{\nabla} x_i dS = \int_B p n_i dS + \int_B (\hat{n} \times \vec{\omega}) \cdot \vec{\nabla} x_i dS \tag{S5}$$

Subtracting (S4) from (S5) we get

$$\begin{aligned} F_B^i = &-\rho \int_{V_f} \frac{\partial \vec{u}}{\partial t} \cdot \vec{\nabla}\Phi^{(i)} dV - \rho \int_{V_f} (\vec{\omega} \times \vec{u}) \cdot \vec{\nabla}\Phi^{(i)} dV - \int_B \frac{1}{2}\rho U^2 n_i dS \\ &+ \mu \int_B (\vec{\omega} \times \hat{n}) \cdot \vec{\nabla}(\Phi^{(i)} - x_i) dS + \mu \int_\Sigma (\vec{\omega} \times \hat{n}) \cdot \vec{\nabla}\Phi^{(i)} dS \end{aligned} \tag{S6}$$

The unsteady term in the above equation can be expanded as follows:

$$\begin{aligned} &\rho \int_{V_f} \frac{\partial \vec{u}}{\partial t} \cdot \vec{\nabla}\Phi^{(i)} dV = \rho \int_{V_f} \vec{\nabla} \cdot \left(\frac{\partial \vec{u}}{\partial t} \Phi^{(i)} \right) dV = \rho \int_{B+\Sigma} \hat{n} \cdot (\frac{\partial \vec{u}}{\partial t} \Phi^{(i)}) dS \\ =&\rho \int_{B+\Sigma} \hat{n} \cdot \frac{d\vec{u}}{dt} \Phi^{(i)} dS - \rho \int_{B+\Sigma} \hat{n} \cdot (\vec{\omega} \times \vec{u}) \Phi^{(i)} dS - \rho \int_{B+\Sigma} \hat{n} \cdot \frac{1}{2} \vec{\nabla} u^2 \Phi^{(i)} dS \\ =&\rho \int_{B+\Sigma} \hat{n} \cdot \frac{d\vec{u}}{dt} \Phi^{(i)} dS - \rho \int_{V_f} \vec{\nabla} \cdot \left[(\vec{\omega} \times \vec{u}) \Phi^{(i)} \right] dV - \rho \int_{V_f} \vec{\nabla} \cdot \left(\frac{1}{2} \vec{\nabla} u^2 \Phi^{(i)} \right) dV \end{aligned} \tag{S7}$$

Substituting (S7) into (S6) and using (S2), the force exerted on the immersed body can be expressed as follows:

$$
\begin{aligned}
F_B^i = & \underbrace{-\rho \int_B \hat{n} \cdot \frac{d\vec{U}}{dt} \Phi^{(i)} dS}_{\text{(i)}} \underbrace{-\rho \int_B \frac{1}{2} U^2 \hat{n} \cdot \vec{\nabla} \Phi^{(i)} dS}_{\text{(ii)}} \underbrace{+\mu \int_B (\vec{\omega} \times \hat{n}) \cdot \vec{\nabla} \left(\Phi^{(i)} - x_i \right) dS}_{\text{(iii)}} \\
& \underbrace{+\rho \int_{V_f} \vec{\nabla} \cdot \left[(\vec{\nabla} \frac{1}{2} u^2) \Phi^{(i)} \right] dV}_{\text{(iv)}} \underbrace{+\rho \int_{V_f} \left[\vec{\nabla} \cdot (\vec{\omega} \times \vec{u}) \right] \Phi^{(i)} dV}_{\text{(v)}} \\
& \underbrace{-\rho \int_{\Sigma} \hat{n} \cdot \frac{d\vec{v}'}{dt} \Phi^{(i)} dS}_{\text{(vi)}} \underbrace{+\mu \int_{\Sigma} (\vec{\omega} \times \hat{n}) \cdot \vec{\nabla} \Phi^{(i)} dS}_{\text{(vii)}}
\end{aligned}
\tag{S8}
$$

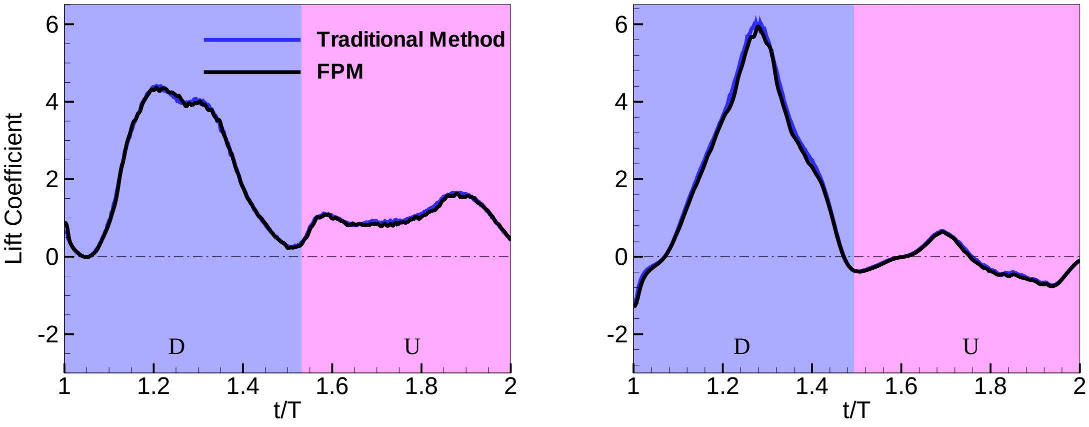

(a)

(b)

Figure S2. Comparison of lift calculation by the traditional method (blue line) and the Force Partitioning Method (FPM) (black line) in (a) Hovering Hawkmoth flight at Re=1000 and (b) Fruit fly at Re=100 throughout a full stroke cycle.

It is noted that terms (i)-(iii) in the above equation are surface integrals over the immersed body involving velocity and vorticity, terms (iv) and (v) are volume integrals involving velocity and vorticity, and terms (vi) and (vii) are surface integrals over the outer boundary. equation. By decomposing the flow velocity into potential ($\vec{u}_\phi$) and rotational ($\vec{u}_v$) components via the Helmholtz decomposition (see S3), term (iv) can be further split into two

terms, leading to the final expression for the FPM as shown in Eq. (4).

$$F_B^i = F_\kappa^i + F_\omega^i + F_\sigma^i + F_\phi^i + F_\Sigma^i \quad \text{for } i = 1,2,3; \quad \text{where} \tag{S9a}$$

$$F_\kappa^i = \underbrace{-\rho \int_B \frac{d\vec{U}}{dt} \cdot \hat{n}\Phi^{(i)} dS}_{F_{\kappa_\mathrm{I}}^{(i)}} \underbrace{-\rho \int_B \frac{1}{2} U^2 \hat{n} \cdot \vec{\nabla}\Phi^{(i)} dS}_{F_{\kappa_\mathrm{II}}^{(i)}} \tag{S9b}$$

$$F_\omega^i = \rho \int_{V_f} \vec{\nabla} \cdot \left[(\vec{\omega} \times \vec{u}) + \vec{\nabla} \left(\frac{1}{2} u_v^2 + (\vec{u}_\phi \cdot \vec{u}_v) \right) \right] \Phi^{(i)} dV \tag{S9c}$$

$$F_\sigma^i = \mu \int_B (\vec{\omega} \times \hat{n}) \cdot \vec{\nabla} \left(\Phi^{(i)} - x_i \right) dS \tag{S9d}$$

$$F_\phi^i = \rho \int_{V_f} \vec{\nabla} \cdot \left[\left(\vec{\nabla} \frac{1}{2} u_\phi^2 \right) \Phi^{(i)} \right] dV \tag{S9e}$$

$$F_\Sigma^i = -\rho \int_\Sigma \hat{n} \cdot \frac{d\vec{v}'}{dt} \Phi^{(i)} dS + \mu \int_\Sigma (\vec{\omega} \times \hat{n}) \cdot \vec{\nabla}\Phi^{(i)} dS \tag{S9f}$$

The total force computed from the traditional method, i.e. by integrating the pressure and shear on the surface (see Eq. (1)) should in principle, match that obtained by applying FPM to the computed velocity and vorticity field. However, we note that Eq. (S9) contains derivative terms above and beyond those in the Navier-Stokes equations (such as those in F_ω) that might be subject to truncation errors when evaluated using finite-difference methods. In the current study, we have evaluated all terms in Eq. (S9) using a second-order central-difference scheme, which is consistent with the underlying flow solver. In S2 Fig. we compare the time-variation of the total lift obtained by the traditional method with that obtained from FPM for both cases and it noted that the differences are extremely small. This not only provides a consistency check for the FPM but also indicates that any discretization errors associated with the implementation of the FPM are negligible.

It is further noted that F_{κ_II} is identically equal to zero at each time-instant for a zero thickness membrane such as the insect wings that are modeled here since the contribution to the surface integral from one side of the wing cancels out the contribution from the other side. Furthermore, dimensional analysis indicates that for a compact body in a three-dimensional domain, $F_\Sigma \sim O\left(\frac{1}{r^3}, \frac{\mu}{r^5}\right)$ where r is the distance between the body and the outer boundary of the domain. Thus, a sufficiently large domain ensures that this term does not have a significant contribution to the force. Indeed, for the domain sizes employed in the current simulations, the root-mean-square of the lift coefficient associated with this term is at most $\mathrm{O}\left(10^{-4}\right)$, which is negligible compared to the O(1) magnitude of the total lift. Similarly, the F_ϕ lift coefficient is also found to be $\mathrm{O}\left(10^{-5}\right)$, and therefore negligible.
