# Supplementary material for: Centripetal Acceleration Reaction: An Effective and Robust Mechanism for Flapping Flight in Insects
Source: PLoS One. 2015 Aug 7;10(8):e0132093. doi: 10.1371/journal.pone.0132093 (PMC4529139; doi:10.1371/journal.pone.0132093)
Supplement: S3 Text — (PDF) [file pone.0132093.s006.pdf]

S3 Helmholtz Decomposition

Using the Helmholtz velocity decomposition [6], the velocity vector field can be decomposed as follows:

$$\vec{u} = \vec{u}_\phi + \vec{u}_v \tag{S10}$$

where $\vec{u}_\phi = \vec{\nabla}\phi$ and $\vec{u}_v = \vec{\nabla} \times \vec{A}$ correspond to the curl-free (potential) and rotational components of the velocity field respectively, with ϕ and A corresponding to the velocity and vector potentials, respectively. In the above decomposition, the potential ϕ may be obtained by solving a Laplace equation for ϕ and $\vec{u}_v$ from the Biot-Savart law (i.e. $\vec{u}_v(\vec{r}) = \frac{1}{4\pi}\int_V \frac{\vec{\omega}(\vec{r}')\times(\vec{r}-\vec{r}')}{|\vec{r}-\vec{r}'|^3}dV'$, where $\vec{\omega}$ is the vorticity in the flow) but here we apply the above decomposition to the Eq. (2) to obtain the following decomposition

$$\rho\frac{\partial \vec{u}_\phi}{\partial t} + \frac{1}{2}\rho\vec{\nabla}\left(\vec{u}_\phi \cdot \vec{u}_\phi\right) = -\vec{\nabla}p_\phi \tag{S11a}$$

$$\rho\frac{\partial \vec{u}_v}{\partial t} + \rho\vec{\omega}\times\vec{u} + \frac{1}{2}\rho\vec{\nabla}\left(\vec{u}_v \cdot \vec{u}_v + 2\vec{u}_\phi \cdot \vec{u}_v\right) = -\vec{\nabla}p_v - \mu\vec{\nabla}\times\vec{\omega} \tag{S11b}$$

where p_ϕ and p_v are the pressure fields corresponding to the potential flow and the vortical components respectively.

The boundary conditions for the two equations above require careful consideration. If the velocity of the wing surface $\vec{U}$ is decomposed into $\vec{U} = U_n\hat{n} + U_t\hat{t}$ where U_n and U_t are the components normal and tangential to the surface respectively, then the boundary condition $\vec{U}_\phi \cdot \hat{n} = U_n$ on the wing surface ensures that $\vec{u}_\phi$ is precisely the potential flow associated with this configuration and Eq. (S11a) with this boundary condition is the well-known Euler equation. The solution of the potential flow equations however, produces a slip velocity on the surface and the total potential flow velocity on the surface therefore is $\vec{U}_\phi = U_n\hat{n} + U_{\phi_t}\hat{t}$ where U_{ϕ_t} is the potential slip velocity. For condition (S10) to hold, the surface boundary condition for the viscous component is $\vec{U}_v = \left(U_t - U_{\phi_t}\right)\hat{t}$. Thus, the velocity of the surface of the wing B can also be written as

$$\vec{U} = \vec{U}_\phi + \vec{U}_v \tag{S12}$$

where we identify $\vec{U}_\phi$ as the potential flow velocity on the surface and $\vec{U}_v$ the tangential component of surface velocity associated with the viscous component of the flow.
