# Supplementary material for: Centripetal Acceleration Reaction: An Effective and Robust Mechanism for Flapping Flight in Insects
Source: PLoS One. 2015 Aug 7;10(8):e0132093. doi: 10.1371/journal.pone.0132093 (PMC4529139; doi:10.1371/journal.pone.0132093)
Supplement: S4 Text — (PDF) [file pone.0132093.s007.pdf]

S4 Force on a Flapping Wing in Potential Flow

By applying FPM to the Euler equation (S11a) we obtain the net force on the wing due to potential flow to be

$$
\begin{aligned}
F_B^i = &-\rho \int_{B+\Sigma} \hat{n} \cdot \frac{d\vec{U}_\phi}{dt} \Phi^{(i)} dS - \rho \int_B \frac{1}{2} U_\phi^2 n_i dS \\
&+\rho \int_{V_f} \vec{\bigtriangledown} \cdot [(\vec{\bigtriangledown} \frac{1}{2} u_\phi^2) \Phi^{(i)}] dV
\end{aligned}
\tag{S13}
$$

For a wing undergoing periodic motion in an inviscid potential flow, since the flow field at the beginning and end of the flapping cycle is *exactly* the same, this necessarily implies that the wing does not transfer any net momentum to the flow over one wing flap. From this it follows that the cycle-averaged force associated with potential flow for a flapping wing, which is given by the above equation, is necessarily zero.
