# Supplementary material for: Centripetal Acceleration Reaction: An Effective and Robust Mechanism for Flapping Flight in Insects
Source: PLoS One. 2015 Aug 7;10(8):e0132093. doi: 10.1371/journal.pone.0132093 (PMC4529139; doi:10.1371/journal.pone.0132093)
Supplement: S5 Text — (PDF) [file pone.0132093.s008.pdf]

## S5 References and Notes


1. Wu, J. Z., Pan, Z. L. & Lu, X. Y. (2005). *Unsteady fluid-dynamic force solely in terms of control-surface integral.* Phys. Fluids 17:098102.

2. Wu, J. Z., Lu, X. Y. and Zhuang, L. X. (2007) *Integral force acting on a body due to local flow structures.* J. Fluid Mech. 576:265-286.

3. Quartapelle, L., Napolitano, M. (1983). *Force and Moment in Incompressible Flows.* AIAA Journal 21:911-913.

4. Howe, M. S. (1995). *On the force and moment exerted on a body in an incompressible flow.* Q. J. Mech. Appl. Maths 48:401-426.

5. Magnaudet, J (2011). *A 'reciprocal' theorem for the prediction of loads on a body moving in an inhomogeneous flow at arbitrary Reynolds number* J. Fluid Mech. 689:564-604.

6. Batchelor, G. K. (1967). *An Introduction to Fluid Dynamics* Cambridge University Press.
